# The sensing of mitochondrial DAMPs by non-immune cells

**DOI:** 10.15698/cst2019.06.190

**Published:** 2019-05-23

**Authors:** Aida Rodríguez-Nuevo, Antonio Zorzano

**Affiliations:** 1 Institute for Research in Biomedicine (IRB Barcelona). The Barcelona Institute of Science and Technology, Barcelona, Spain.; 2 Departament de Bioquímica i Biomedicina Molecular, Facultat de Biologia, 08028 Barcelona, Spain.; 3 CIBER de Diabetes y Enfermedades Metabólicas Asociadas (CIBERDEM), Instituto de Salud Carlos III.

**Keywords:** mitochondria, DAMP, immunity, mitochondrial DNA, TLR9, cGAS

## Abstract

Mitochondria are the source of damage-associated molecular patterns (DAMPs), which are molecules that play a key modulatory role in immune cells. These molecules include proteins and peptides, such as N-formyl peptides and TFAM, as well as lipids, and metabolites such as cardiolipin, succinate and ATP, and also mitochondrial DNA (mtDNA). Recent data indicate that somatic cells sense mitochondrial DAMPs and trigger protective mechanisms in response to these signals. In this review we focus on the well-described effects of mitochondrial DAMPs on immune cells and also how these molecules induce immunogenic responses in non-immune cells. Special attention will be paid to the response to mtDNA.

## MITOCHONDRIA ARE IMMUNOGENIC ORGANELLES

The efficiency of the innate immune system is determined by the capacity of distinct cell types to discriminate self from non-self structures. The dysregulation of this ability results in either immunodeficiency pathologies or autoinflammatory and autoimmune diseases. The immune system is primed to recognize pathogen-associated molecular patterns (PAMPs), derived from infection, through a variety of receptors. However, misplaced self-molecules can also trigger similar types of responses. Such molecules are called damage-associated molecular patterns (DAMPs). Mitochondria produce DAMPs and in fact, are relevant contributors to the cellular generation of these damage signals. In this regard, both the origin and features of these molecules account for the immunogenic capacity of the mitochondrion.

Mitochondria arose around two thousand million years ago, which makes them one of the most ancient endomembrane systems in eukaryotic cells. In 1967, Lynn Margulis rescued the long forgotten endosymbiont theory of organelle origin [1]. This proposes that eukaryotic cells derived from the engulfment of an α-proteobacterium by the eukaryotic progenitor. The resemblance of modern mitochondria to their bacterial ancestor supports this theory. Among other features, mitochondria are comprised of two functionally different and separate membranes that surround a matrix compartment that contains the unmethylated mitochondrial circular genome, which is organized as nucleoids throughout the matrix [2]. The bacteriallike characteristics of mitochondria also reinforce the notion of them being hubs of immunity. The proteins found in mitochondria are structurally similar to those in bacteria and enable their recognition by the same receptors of the immune system [3].

Mitochondria are pivotal organelles for many cellular functions and are the primary energy-generating system in most eukaryotic cells. The architecture of mitochondria is essential for their proper function and also for the confinement of mitochondria-derived immunogenic molecules. At the ultrastructural level, mitochondria are comprised by two membranes, namely the outer mitochondrial membrane (OMM) and the inner mitochondrial membrane (IMM). The OMM is structurally simple and highly permeable to small molecules and ions, while at the same time it protects the cell from noxious mitochondrial products, including reactive oxygen species (ROS), immunogenic mtDNA [4] and death signals. The IMM is morphologically more complex, and it creates an impermeable barrier between the matrix and the intermembrane space. This restrictive permeability and proper cristae morphology are the two major physical features that enable mitochondria to perform oxidative phosphorylation [5]. Additionally, these organelles participate in intermediary metabolism, the regulation of programmed cell death, calcium homeostasis, and the generation and control of ROS [2, 6, 7]. Mitochondrial functionality is synonymous with cellular homeostasis. In this regard, diverse molecules extruded from mitochondria alert neighboring cells, the immune system, and the producing cell itself about mitochondrial dysfunction. This signal triggers various mechanisms aimed to revert the defect and recover homeostasis, or, under chronic or more severe conditions, to induce a systemic response.

## MITOCHONDRIA GENERATE DIFFERENT TYPE OF IMMUNOGENIC MOLECULES OR DAMPs

Mitochondria-derived DAMPs (hereafter MTDs) include not only proteins but also DNA, lipids and metabolites, and they show immunogenic capacity when misplaced or imbalanced. In this review we focus on the well-described effects of MTDs on immune cells and also how these molecules induce immunogenic responses in non-immune cells.

MTDs are recognized by Pattern Recognition Receptors (PRRs). Usually, the receptors that recognize specific mitochondrial molecules are those that engage to the pathogenic homologs of these molecules. Therefore, the response triggered by MTDs resembles pathogenic effects. Hence, MTDs in sterile conditions have been studied with the aim to unravel autoimmunity and autoinflammatory diseases.

**Figure 1:**
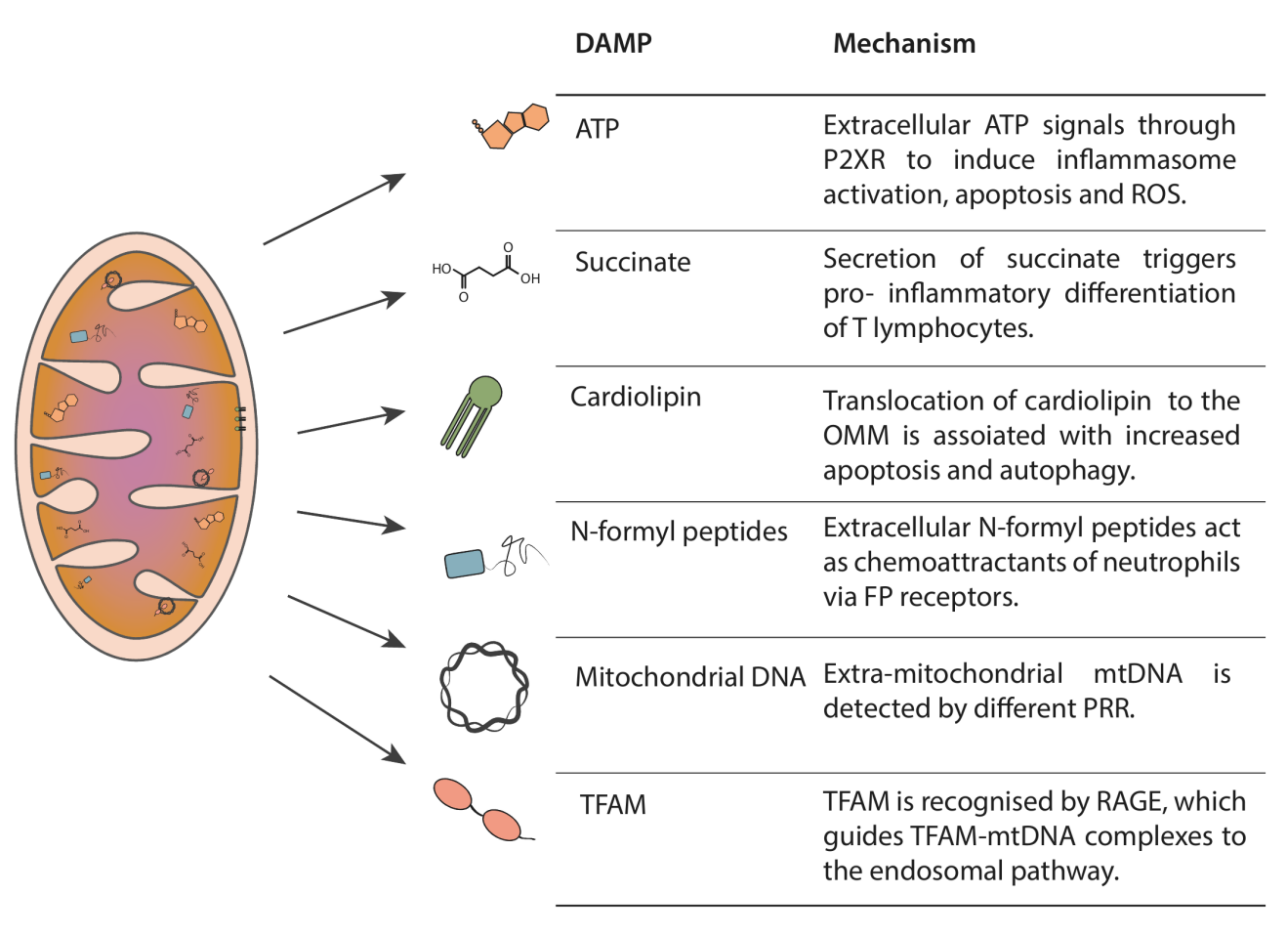
FIGURE 1: Mitochondria-derived DAMPs. Mitochondria generate immunogenic molecules, named damage associated molecular patters (DAMPS). The scheme represents the localization of the different DAMPs in their non-immunogenic state, as well as a brief description of their immunogenic capacity when misplaced.

Mitochondria produce several DAMPs, such as ATP, succinate, cardiolipin, N-formyl peptides, mtDNA and mitochondrial transcription factor A (TFAM), which serve as danger flags for immunological signaling (**Figure 1**) [4, 8–11].

In addition to being the source of DAMPs, mitochondria are also linked to immunity through their role as innate immune platforms that harbor the mitochondrial antiviral-signaling protein (MAVS) as a viral RNA sensor and the Nod-like receptor 3 (NLRP3) inflammasome as a multiple immunogenic receptor [12–16].

### Adenosine triphosphate

ATP is the energetic currency in all living organisms. It is synthesized mainly in mitochondria by ATP synthase coupled to the electron transport chain in the IMM. It is transported to the cytosol by the ATP-ADP translocase and used by many energy-demanding reactions in the cell. The conversion from adenosine triphosphate to adenosine diphosphate through the donation of a phosphate group enables biochemical reactions.

In addition to the crucial role of intracellular ATP as an energy source for the maintenance of cellular homeostasis, it is also a key player in extracellular signaling. ATP is released from cells by cell damage, exocytosis as well as by non-vesicular mechanisms, which include ATP release channels [17]. It has been demonstrated that extracellular ATP is sensed by P2XR and P2YR receptors in the plasma membrane [18, 19]. ATP is secreted from various types of cells under stress conditions. For instance, bacterial, hypotonic and mechanical insults on epithelial cells result in increased secretion of ATP [20–22]. High levels of extracellular ATP are sensed by leukocytes and promote relaxation, vasodilatation, neurotransmission, platelet aggregation, ion transport regulation, cell growth, and immune response—all processes triggered in response to tissue damage [23–25]. In particular, neutrophils activate chemotaxis, release IL-8 and elastase, increase adhesion capacity to endothelial cells, cause degranulation, and produce ROS and ATP to further enhance the innate immune response [26]. In monocytes and macrophages, extracellular ATP promotes the production of the pro-inflammatory cytokines IL-1β and IL-18. ATP binds to P2X7 receptor and induces K^+^ efflux through the P2X7 channel. This results in caspase-1 cleavage in the NLRP3 inflammasome, which in turn promotes cytokine maturation and secretion [27, 28]. Both in monocytes and T lymphocytes, ATP induces shedding of L-selectin by P2X7R activation, thereby leading to transmigration through the endothelium. These observations thus indicate the involvement of ATP in both innate and adaptive immunity [29].

### Succinate

Succinate is an intermediate of the tricarboxylic acid (TCA) cycle that is generated from succinyl-CoA via succinyl-CoA ligase. However, succinate has been shown to be secreted to the extracellular media *in vitro*, and this is stimulated by antimycin A treatment, which inhibits electron transfer between cytochrome b and c1 [30]. Indeed, extracellular succinate acts as a signaling molecule and is recognized by immune cells through its G-protein-couple receptor, namely succinate receptor 1 (SUCNR1, also named GPR91) [31]. The activation of the receptor stabilizes hypoxia-inducible factor-1 alpha (HIF-1α), which favors the pro-inflammatory differentiation of T lymphocytes [32]. Succinate is also described to have synergic effects with Toll-like receptor (TLR) ligands in dendritic cells for the production of cytokines.

### Cardiolipin

Cardiolipin (CL) is a phospholipid that accounts for 20% of total lipid content in the IMM [33–35]. CL is composed of two phosphatidylglyceride backbones and a glycerol head group. Four fatty acids chains, with different lengths and degrees of saturation, are bound to CL [36]. This phospholipid is pivotal in many mitochondrial processes, including protein import, dynamics, respiratory chain functionality, and metabolism [37, 38]. Cellular necrosis exposes CL to the extracellular media, which can be sensed by T cells through CD1d [39]. Also, CL can bind directly to NLRP3 and activate inflammasome-mediated immune response [40]. CL is increased in tracheal aspirates of human pneumonia patients, as well as in lung injury models [41]. However, like other MTDs, CL is found in both bacterial and mitochondrial membranes. Therefore, to date, it has been difficult to verify the origin of pathology-associated high levels of this phospholipid in the extracellular media.

### N-formyl peptides

Bacteria use the addition of a formyl group, a carbonyl bonded to hydrogen, to methionine to initiate protein synthesis. Bacterial N-formyl peptides (NFPs) serve as chemoattractants to activate host phagocytes [42]. Polymorphonuclear and mononuclear phagocytes show high expression of formyl peptide receptors (FPRs), members of the seven families of transmembrane G protein-couple receptors [43]. Recognition of NFPs by FPR in the plasma membrane of the phagocyte initiates various defense responses of the immune cell, such as morphological polarization, locomotion, phagocytosis, ROS generation, and protease secretion [42]. NFPs were first described to present chemoattractant capacity for neutrophils and platelets [44, 45]. Mice unable to detect NFPs, by genetic knockout of formyl peptide receptor 1, show higher susceptibility to infection by *Listeria monocytogenes* [46]. In humans, localized juvenile periodontitis patients carry dysfunctional variant alleles of the FPR gene and present reduced neutrophil chemotaxis capacity to NFPs [47, 48]. Interestingly, NFPs are extruded not only by pathogens like *Escherichia coli* but also by the mitochondria of damaged or dying cells. Mitochondrial formylation of methionine is needed for translation initiation of mRNA transcribed from mtDNA, a mechanism reminiscent of bacteria [49]. Thus, mitochondria produce NFP due to the translation of mtDNA-encoded proteins within the mitochondrial matrix. Mitochondrial extracts of NFPs induce the chemotaxis of polymorphonuclear cells, whereas the non-formylated peptides are innocuous. Moreover, NFPs are secreted only by necrotic cells, thus excluding apoptotic cells [45]. These observations suggest that NFPs have dual roles in tissue homeostasis. On the one hand, they promote the clearance of bacteria and infected cells. On the other hand, they enable the identification of damage cells undergoing necrosis, a process that will lead to extrusion of mitochondrial content. NFPs therefore allow the rapid clearance of these damaged cells by phagocytes.

### Mitochondrial DNA

The mitochondrial genome is a double-stranded circular DNA molecule of around 16 kilobases, present in hundreds to thousands of copies per cell. It is packed with nucleoids, which are slightly elongated, irregularly shaped structures of approximately 80–100 nm. Nucleoids associate with the IMM and distribute throughout mitochondria [50]. They contain relatively high levels of TFAM (1 subunit every 16–17 bp of mtDNA), which is essential for mtDNA maintenance because it is responsible for mtDNA packaging [51–56]. There is some debate regarding the number of mtDNA copies per nucleoid. While some authors have described several copies of mtDNA per nucleoid [57], others have reported only one copy [56, 58]. The organization of mtDNA into nucleoids is essential for the correct distribution and segregation of mtDNA. The mitochondrial genome encodes for 22 tRNA, 2 rRNA and 13 essential subunits of the mitochondrial oxidative phosphorylation system: complex I (ND1—ND6), complex III (Cyt b), complex IV (COX I—COX III), and complex V (A8 and A6). It also contains a few non-coding sequences, the largest being the displacement loop (D-loop). This region of the mtDNA is also denominated the control region, because it contains the promoter for the transcription of both light and heavy strands (LSP and HSP) and the origin of replication for the heavy strand [59, 60].

Owing to the α-proteobacterial origin of mitochondria, mtDNA has unique features that are important for its role in innate immune responses and inflammation. Misplaced mtDNA has been widely shown to induce a proinflammatory state [4, 61–65]. Methylation of the CpG regions in mtDNA differs to that of the nuclear DNA and confers mtDNA with immunogenic potential due to its resemblance to DNA of pathogens, specially bacteria. Some authors have described the absence of methylation in mtDNA, while others report that the nuclear DNA methyltransferase (DNMT1) is found in mitochondria, thereby suggesting some degree of methylation in mammalian cells [66, 67]. In addition to hypomethylation, mtDNA can undergo oxidative damage, which is immunogenic and can be recognized by PRRs, independently of the degree of methylation [68, 69]. Extracellular mtDNA binds TLR9, which has also been described to recognize unmethylated or undermethylated CpG DNA. In macrophages, mtDNA engagement with TLR9 induces the production of proinflammatory cytokines, chemotaxis and phagocytic activation through a MyD88-dependent signaling cascade. TLR9 has been associated with the development of polymicrobial acute kidney injury through the recognition of mtDNA release [70].

High levels of circulating mtDNA have been linked to liver dysfunction and increased neutrophil-mediated inflammatory responses [71]. The injection of mtDNA induces lung injury and arthritis with infiltration of mononuclear cells in mice [72]. These data support the notion that mtDNA participates in the development of inflammatory responses *in vivo* [4, 73]. mtDNA also induces inflammation in microglial and neuronal cells or in mouse brains [74, 75]. Furthermore, it has been reported that mtDNA stress elicited by TFAM deficiency triggers cytosolic antiviral signaling, promoted by cytosolic mtDNA leakage [64]. Thus, the degree of packaging, the stability, the localization and the presence of oxidative damage modifications or mutations are implicated in mtDNA-innate immune signaling [12].

### TFAM

Mitochondrial transcription factor A (TFAM) is member of the HMG box family of proteins. TFAM interacts with mitochondrial DNA and regulates both its transcription and replication, thus modulating mtDNA-encoded protein expression and mtDNA copy number [76, 77]. TFAM correct expression is crucial for mitochondrial function and thus cellular homeostasis [55]. The presence of extracellular TFAM is described to induce an inflammatory response, similarly to the action of another DAMP of the same family of proteins, namely HMGB1. TFAM also enhances the immunogenicity of mtDNA [78]. While bound to mtDNA, it can interact with the plasma membrane receptor RAGE and induces the internalization of mtDNA, thereby promoting its recognition by TLR9 [79]. Also, TFAM enhances cytokine secretion in combination with NFPs [80]. Treatment with TFAM increases the levels of Il6 and TNF in the serum of rats and in the media from RAW264.7 macrophage cultures [81].

## NON-IMMUNE CELLS ALSO RESPOND TO MITOCHONDRIAL DAMPs

Mitochondrial DAMPs were initially reported in cells of the immune system [82]. However, we now know that somatic cells also respond to mitochondrial DAMPs to either trigger protective mechanisms or pathways to exacerbate the signal. In this regard, the release of mitochondrial DAMPs in ischemia/reperfusion during liver transplantation, together with pro-inflammatory cytokines, causes hepatic inflammation and cell death [83]. In contrast, some mitochondrial DAMPs may have a protective role. The activation of the P2X7 receptor, an ATP-gated trimeric membrane cation channel, induces plasmalemmal blebbing [84], which prevents cellular damage triggered by bacterial pore-forming toxins. On the basis of these data, it has been proposed that ATP modulates inflammation and prevents cell death upon activation of the P2X7 receptor.

Necrosis is not strictly necessary for the release of mitochondrial DAMPs. Duregatti and colleagues reported that neurons treated with presynaptic toxins release hydrogen peroxide, as well as mtDNA and cytochrome c, secondary to mitochondrial dysfunction. These DAMPs activate Schwann cells to initiate neural degeneration. The release of mtDNA and cytochrome c results from the opening of the mitochondrial transition pore, and the maintenance of mitochondrial permeability was shown to be key to restraining mitochondrial DAMP release [85].

Cardiolipin is asymmetrically enriched in the IMM. Under stimuli determining cell or mitochondrial dysfunction such as membrane depolarization or tBid binding, CL is translocated to the OMM, a process facilitated by the phospholipid scramblase 3 (PLS3) [86]. The presence of CL in the OMM induces mitophagy [87], and apoptosis by reducing OMM permeability and thus enabling cytochrome c release. Regarding the immunogenic capacity of CL, it acts as a signaling platform in the recruitment of inflammasome particles and to induce the activation of these molecules [86, 88].

Mitochondrial DAMPs can play modulatory roles, for example, by increasing endothelial cell permeability, thus allowing the transmission of the immunogenic response to distal organs [89]. These studies indicate that mitochondrial DAMPs are important in the different levels of the immunogenic response in non-immune cells.

The regulatory role of succinate or N-formyl peptides in non-immune cells remains unclear. However, given that the expression of the succinate receptor, SUCNR1, is high in liver and kidney [90], it is likely that it mediates mitochondrial stress in these tissues. Similarly, N-formyl peptide receptors FPR1 and FPR2 show a relative broad expression so they may also play a modulatory role in various tissues [91].

## MECHANISMS OF RESPONSE TO MITOCHONDRIAL DNA

Since nucleic acids are central for the replication and propagation of most pathogens, it is not surprising that their detection is covered by various kinds of PRRs localized in diverse cellular compartments. In particular, mtDNA is recognized by four innate immune receptors: cytosolic cyclic GMP-AMP synthase (cGAS), endosomal TLR9, and the two inflammasomes: Absent In Melanoma 2 (AIM2), and NOD, LRR and Pyrin domain-containing protein 3 (NLRP3) [92, 93]. Here we focus mainly on cGAS and TLR9 as mtDNA sensors since the mechanism through which mtDNA activates inflammasomes is poorly characterized.

### Mitochondrial DNA and cGAS signaling

**Figure 2:**
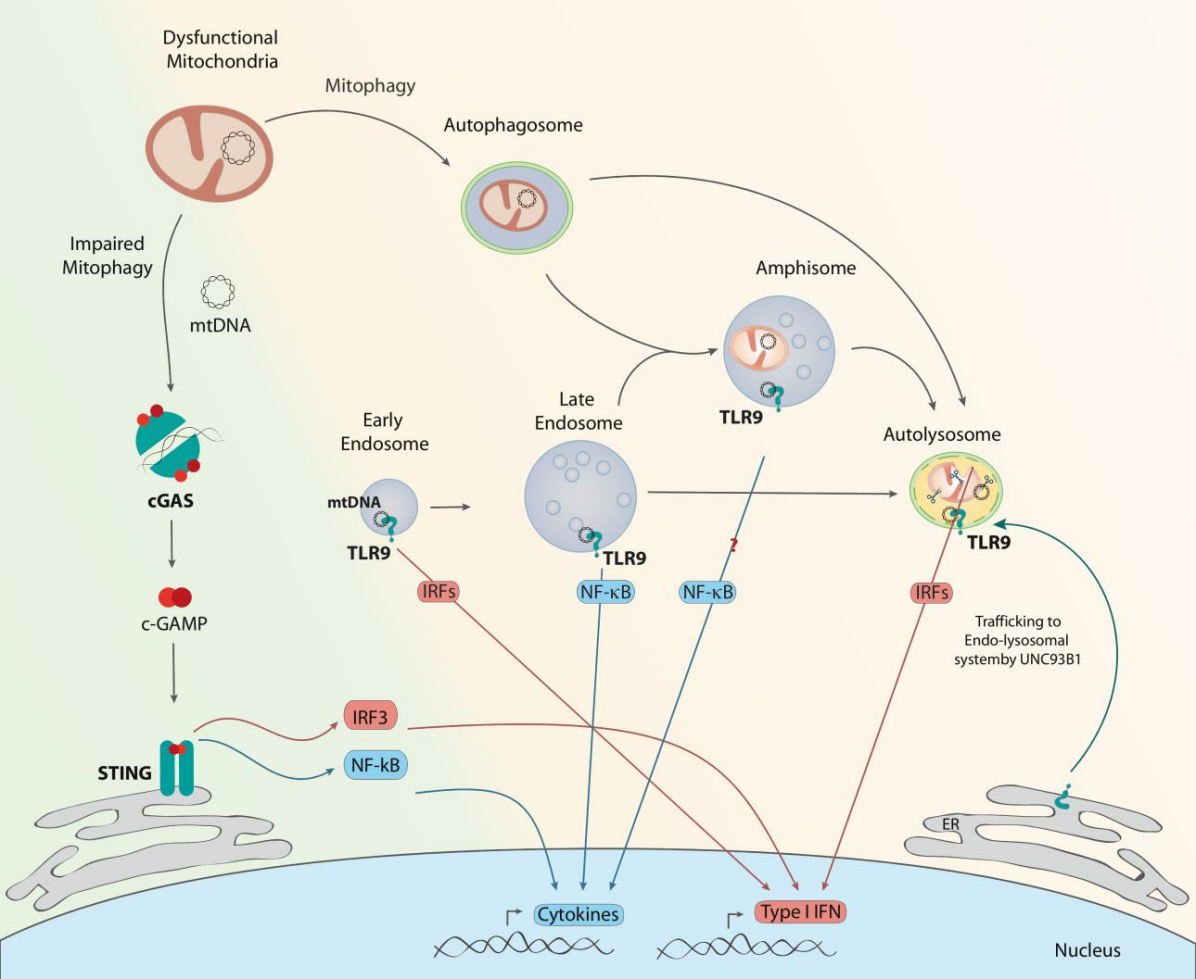
FIGURE 2: cGAS and TLR9 activation by mtDNA. Under conditions of impaired mitophagy initiation (green background), thus accumulation of dysfunctional mitochondria, there is mtDNA leakage to the cytosol. cGAS homodimers recognize double stranded DNA as mtDNA and produce c-GAMP, which interacts with STING, triggering IRF3 or NF-κB, and in turn the expression of type I IFNs or cytokines, respectively. Under conditions in which mitophagosomal formation occurs normally but resolution is defective (orange background), mtDNA instability can lead to its recognition by TLR9 in different endosomal compartments. TLR9 is recruited from the ER to the endo-lysosomal system, guided by UNC93B1. Engagement of TLR9 in early endosomes or lysosomes results in type I IFN expression through various IRFs. Interaction of mtDNA with TLR9 in late endosomes results in NF-κB activation. Given the resemblance of amphisomes to late endosomes, TLR9 engagement in amphisomes could result in cytokine production.

cGAS is the most recently described DNA sensor [94, 95]. Cytosolic double stranded (ds)DNA activates cGAS to form a dimeric cGAS-DNA complex that synthesizes cyclic GMPAMP or cGAMP from ATP and GTP. This cGAMP functions as a second messenger because it is a high-affinity ligand for the endoplasmic reticulum (ER) membrane adaptor protein stimulator of interferon genes (STING) [96]. cGAMP induces conformational changes in STING, which results in the subsequent activation of the transcription factors NFκB and IRF3 through the kinases IKK and TBK1, respectively (**Figure 2**) [97–101]. Interestingly, cGAS induces autophagy independently of STING as a protective mechanism against ischemia-reperfusion injury in liver [102].

Under conditions in which DNA is bound to HMGB1 or TFAM and forms a protein-DNA ladder, cGAS signaling is promoted [103]. Specifically, mtDNA has been reported to trigger a type I interferon (IFN) response and expression of IFN-stimulated genes (ISG) in a *Tfam* heterozygous context [64]. West and colleagues observed mtDNA stress, characterized by reduced nucleoid number and increased nucleoid size. In these conditions, mtDNA was found in the cytosolic fraction in the context of mitochondrial hyperfusion. Interestingly depletion of mitochondrial fusion protein Mfn1 normalizes ISG expression. In obesity-induced insulin resistance, the release of mtDNA to the cytosol has been described as a major driver of the chronic inflammation associated with the disease, through the activation of the cGAS-STING pathway [104]. This pathway is also activated upon release of mtDNA in apoptotic conditions, although the apoptosis pathway silences the immunogenic response. McArthur and colleagues used apoptosis as a model situation of mtDNA release to the cytosol and found that the BAK/BAX macropore allows the IMM to herniate, creating a protrusion in the mitochondrial surfaces of naked IMM. The loss of membrane integrity then allowed exposure of mtDNA to the cytosol [105].

### Mitochondrial DNA as a TLR9 agonist

TLR9 was the first protein of the TLR family to be described as a nucleic acid sensor (**Figure 2**). It is expressed mainly in immune system cells, including dendritic cells and macrophages. However, it is also found in other non-immune cells such as muscle and epithelial cells, among others [106]. It binds specifically to unmethylated CpG DNA, like the mtDNA, in the endolysosomal compartment [12, 107–109]. TLR9 signals through the myeloid differentiation primary response protein 88 (MyD88), which activates a number of kinases and transcriptional factors, namely mitogen-activated protein kinases (MAPK), nuclear factor-κB (NF-κB) and IRF7 to enhance pro-inflammatory and type I interferon responses, respectively. Nucleic acid-sensing TLRs are not detectable at the cell surface but instead reside within internal compartments. In particular, full-length TLR9 localizes in the ER under unstimulated conditions [110]. More recent studies have identified that, upon stimulation, full-length TLR9 traffics through the Golgi apparatus to the endolysosomal compartment, guided by the accessory protein UNC93B1, and it is then cleaved to become DNA sensing-competent [111–115]. The recruitment of TLR9 to the endolysosomal compartment is key for its function since proteolytic activation of TLR9 occurs in endosomes, and in addition, MyD88 localizes in this compartment.

There is some evidence suggesting that the specific endolysosomal compartment in which the interaction between DNA and TLR9 takes place determines the type of immune response generated [116, 117]. Thus, it has been proposed that TLR9 signaling from late endosomes leads to the activation of NF-κB, whereas TLR9 signaling from a distinct population of endosomes brings about the recruitment of IRF and induction of type I IFN (IFN-a, IFN-b). This alternative compartment is hypothesized to be either early endosomes or lysosomes [118, 119]. Interestingly, TLR9 modulates energy metabolism in cardiomyocytes [120], reduces ATP, and enhances AMPK activity [121]. In all, distinct cellular responses can be elicited in different cell types through the activation of the TLR9 receptor system.

Regarding mtDNA, unmethylated CpG motifs of mtDNA trigger TLR9 signaling. Several studies have reported the relevance of circulating or extracellular mtDNA in TLR9-dependent inflammation in diseases such as rheumatoid arthritis [122], atherosclerosis [10, 123], acute liver injury [71], and *Streptococcus pneumoniae* infections [124], among others [10, 62, 125–129]. Rats subjected to vascular injury showed increased extracellular mtDNA, which led to lung tissue damage through a TLR9-dependent pathway [130]. In pregnancy, high levels of cell death are associated with preeclampsia through a mechanism involving mtDNA and TLR9 [131] Also, high mtDNA levels in non-alcoholic steatohepatitis patients are reported to activate TLR9 and exacerbate the inflammatory profile [129]. Moreover, high mobility group protein B1 (HMGB1), a nuclear DNA-binding protein released from necrotic cells, was found to be an essential component of DNA-containing immune complexes that stimulate cytokine production through a TLR9-MyD88 pathway involving the multivalent receptor RAGE [132]. Similarly, extracellular TFAM-bound mtDNA can induce a further stronger NF-κB activation since plasma membrane receptor RAGE interacts with TFAM and delivers mtDNA to TLR9 [133].

Mitochondrial DNA engages with TLR9 in the lysosomes of DNase II-deficient hearts, thereby suggesting that undegraded mtDNA escaping from autophagy induces TLR9 and causes inflammation in cardiomyocytes [61]. In parallel, De Leo *et al*. reported a non-inflammatory role of the TLR9-DNA interaction in the lysosome cargo response, which is required to sustain the autophagic flux [134]. In skeletal muscle, ablation of the mitochondrial fusion protein Opa1 leads to a severe mitochondrial inflammatory myopathy, which is caused by TLR9 activation through a mechanism that requires mtDNA [135]. This inflammatory process is a primary cell-autonomous response of muscle cells to Opa1 deficiency and it leads to NF-κB activation [135]. Another less characterized response of TLR9 is the interaction with mtDNA and HMGB1 in the cytosol during hypoxia, which is involved in tumor growth [136]. In all, various mechanisms have been implicated in TLR9-associated pathologies. This is not surprising given the multiple factors involved in infection or injury [137–139].

### Inflammasome

Mitochondrial DNA can also be recognized by two members of a superfamily of immunogenic receptors, namely the inflammasomes AIM2 and NLRP3. These are mainly cytosolic multiprotein oligomeric complexes that activate caspase-1, which in turn proteolytically cleaves IL-1β and IL-18, thus generating mature active forms of the secreted cytokines [28]. The inflammasome is triggered not only by pathogen-associated molecular patterns (PAMPs) as flags of pathogen infection but also by DAMPs, which flag cellular malfunction or stress. The activation of the inflammasome involves two sequential signals. The priming signal leads to NF-κB activation, which includes the expression of inflammasome components and inactive forms of the cytokines. This signal prepares the cell for a possible activation of the pathway in response to, for instance, pathological infection. Typically, this first signal is the engagement of a membrane PRR, for example TLR4, but it is also triggered by intracellular receptors like intraluminal TLRs, including TLR7 and TLR9. Experimentally, lipopolysaccharide (LPS) is widely used to prime cells for inflammasome activation.

The second signal is the trigger itself, which interacts with the recognition part of the complex and leads to the oligomerization and activation of the inflammasome particle. In addition to the recognition protein, which is specific for each type of inflammasome, a scaffold protein, the adaptor protein known as ASC, is common to all of them. This protein serves as a bridge from the upstream inflammasome sensor molecule to caspase 1. Inflammasome assembly and oligomerization results in the cleavage of caspase 1, which in turn causes rapid and efficient activation and secretion of large amounts of IL-1β, which were already expressed in response to the priming signal. Inflammasomes fall into several categories. In this regard, Broz and Dixit classified them into the following the groups: 1) the nucleotide-binding oligomerization domain (NOD); the leucine-rich repeat (LRR)-containing protein (NLR) or NLR inflammasomes; 2) the absent in melanoma 2 (AIM2) and pyrin inflammasomes, which are canonical inflammasomes; and 3) non-canonical inflammasomes [140]. NLRP3 is the most studied type of inflammasome, yet the mechanisms of activation are so diverse that there is no consensus regarding whether it has affinity for a wide range of molecules or whether there is an unknown intracellular signal that converges all the different triggers into NLRP3 activation [16, 141, 142]. Classically, the inflammasome has been addressed only in macrophages and other inflammatory cells. However, several studies have reported inflammasome activation in non-immune cells, like podocytes, hepatocytes, and cardiac and skeletal myocytes [143–149]. The involvement of mtDNA in the activation of NLRP3 was reported upon mitochondrial dysfunction, leading to mtDNA leakage to the cytosol in primed macrophages [68]. In fact, mitochondria are proposed to harbour NLRP3 and be able to regulate the activity of the inflammasome complex. On one hand, activation of mitophagy reduces inflammation by clearing mitochondrialbound NLRP3 complexes. On the other hand, mitochondrial ROS can exacerbate inflammasome immunogenic signal [14].

## CONCLUSIONS AND PERSPECTIVES

Mitochondrial DAMPs have been studied mainly in the context of the function of immune cells, and we now know that they play a key role in the activity of the innate immune system, as well as in pathologies associated with immunodeficiencies and autoimmune and autoinflammatory diseases. In this regard, the mechanism by which mitochondrial dysfunction leads to the release of the different DAMPs described and the conditions under which some molecules predominate over others remain elusive. Specific studies are required to clarify this point.

There is a fragmented understanding of the cellular mechanisms that delocalize mtDNA and generate mitochondrial DAMPs. In this context, further research is needed. The relative role of the different cytosolic sensors to mitochondrial DNA should be clarified. In short, a greater knowledge of the stability and targeting of mtDNA and its sensors will allow us to predict the response of given cells to the generation of mitochondrial DAMPs triggered by specific mitochondrial damage.

Finally, future research should seek to unravel how non-immune cells respond to mitochondrial damage caused by the release of DAMPs, and mechanisms involved in these responses. Of particular relevance is understanding those conditions in which the damage to non-immune cells leads to chronic inflammatory responses. The findings of such lines of research would contribute to shedding light on diseases that are only partially understood, as is the case of inflammatory myopathies, and would also help to define pharmacological treatments for the same.

## Abbreviatons:

cGAS: cyclic GMP-AMP synthase
CL: cardiolipin
DAMP: damage-associated molecular pattern
ER: endoplasmic reticulum
FPR: formyl peptide receptor
IFN: interferon
IMM: inner mitochondrial membrane
MTD: mitochondria-derived DAMP
mtDNA: mitochondrial DNA
NFP: N-formyl peptide
OMM: outer mitochondrial membrane
PAMP: pathogen-associated molecular pattern
PRR: Pattern Recognition Receptor
ROS: reactive oxygen species
STING: stimulator of interferon genes
TLR: Toll-like receptor

## References

[1] Sagan L (1967). On the origin of mitosing cells.. J Theor Biol.

[2] Nunnari J, Suomalainen A (2012). Mitochondria: In sickness and in health.. Cell.

[3] Pallen MJ (2011). Time to recognise that mitochondria are bacteria?. Trends Microbiol.

[4] Zhang Q, Raoof M, Chen Y, Sumi Y, Sursal T, Junger W, Brohi K, Itagaki K, Hauser CJ (2010). Circulating mitochondrial DAMPs cause inflammatory responses to injury.. Nature.

[5] Jayashankar V, Mueller IA, Rafelski SM (2016). Shaping the multi-scale architecture of mitochondria.. Curr Opin Cell Biol.

[6] Gunter TE, Buntinas L, Sparagna GC, Gunter KK (1998). The Ca2+ transport mechanisms of mitochondria and Ca2+ uptake from physiological-type Ca2+ transients.. Biochim Biophys Acta.

[7] Hockenbery D, Nunez G, Milliman C, Schreiber RD, Korsmeyer SJ (1990). Bcl-2 is an inner mitochondrial membrane protein that blocks programmed cell death.. Nature.

[8] Krysko D V, Agostinis P, Krysko O, Garg AD, Bachert C, Lambrecht BN, Vandenabeele P (2011). Emerging role of damage-associated molecular patterns derived from mitochondria in inflammation.. Cell.

[9] Nakahira K, Hisata S, Choi AM (2015). The roles of Mitochondrial DAMPs in Diseases.. Antioxid Redox Signal.

[10] Yu EPK, Bennett MR (2014). Mitochondrial DNA damage and atherosclerosis.. Trends Endocrinol Metab.

[11] Arnoult D, Soares F, Tattoli I, Girardin SE (2011). Mitochondria in innate immunity.. EMBO Rep.

[12] West AP, Shadel GS, Ghosh S (2011). Mitochondria in innate immune responses.. Nat Rev Immunol.

[13] Mills EL, Kelly B, O'Neill LAJ (2017). Mitochondria are the powerhouses of immunity.. Nat Immunol.

[14] Zhou R, Yazdi AS, Menu P, Tschopp J (2011). A role for mitochondria in NLRP3 inflammasome activation.. Nature.

[15] Koshiba T, Yasukawa K, Yanagi Y, Kawabata S (2011). Mitochondrial membrane potential is required for MAVS-mediated antiviral signaling.. Sci Signal.

[16] Sutterwala FS, Haasken S, Cassel SL (2014). Mechanism of NLRP3 inflammasome activation.. Ann N Y Acad Sci.

[17] Taruno A (2018). ATP release channels.. Int J Mol Sci.

[18] Sluyter R, Stokes L (2011). Significance of P2X7 receptor variants to human health and disease.. Recent Pat DNA Gene Seq.

[19] Fuller SJ, Stokes L, Skarratt KK, Gu BJ, Wiley JS (2009). Genetics of the P2X7 receptor and human disease.. Purinergic Signal.

[20] Save S, Persson K (2010). Extracellular ATP and P2Y receptor activation induce a proinflammatory host response in the human urinary tract.. Infect Immun.

[21] Hazama A, Shimizu T, Ando-Akatsuka Y, Hayashi S, Tanaka S, Maeno E, Okada Y (1999). Swelling-induced, CFTR-independent ATP release from a human epithelial cell line: lack of correlation with volume-sensitive cl(-) channels.. J Gen Physiol.

[22] Grygorczyk R, Hanrahan JW (1997). CFTR-independent ATP release from epithelial cells triggered by mechanical stimuli.. Am J Physiol.

[23] Deli T, Csernoch L (2008). Extracellular ATP and cancer: an overview with special reference to P2 purinergic receptors.. Pathol Oncol Res.

[24] Sprague RS, Olearczyk JJ, Spence DM, Stephenson AH, Sprung RW, Lonigro AJ (2003). Extracellular ATP signaling in the rabbit lung: erythrocytes as determinants of vascular resistance.. Am J Physiol Heart Circ Physiol.

[25] Burnstock G (2006). Pathophysiology and therapeutic potential of purinergic signaling.. Pharmacol Rev.

[26] Bours MJL, Swennen ELR, Di Virgilio F, Cronstein BN, Dagnelie PC (2006). Adenosine 5'-triphosphate and adenosine as endogenous signaling molecules in immunity and inflammation.. Pharmacol Ther.

[27] Rathinam VAK, Fitzgerald KA (2016). Inflammasome Complexes: Emerging Mechanisms and Effector Functions.. Cell.

[28] Lamkanfi M, Dixit VM (2014). Mechanisms and Functions of Inflammasomes.. Cell.

[29] Jamieson GP, Snook MB, Thurlow PJ, Wiley JS (1996). Extracellular ATP causes loss of L-selectin from human lymphocytes via occupancy of P2Z purinoceptors.. J Cell Physiol.

[30] Shaham O, Slate NG, Goldberger O, Xu Q, Ramanathan A, Souza AL, Clish CB, Sims KB, Mootha VK (2010). A plasma signature of human mitochondrial disease revealed through metabolic profiling of spent media from cultured muscle cells.. Proc Natl Acad Sci U S A.

[31] Rubic T, Lametschwandtner G, Jost S, Hinteregger S, Kund J, Carballido-Perrig N, Schwarzler C, Junt T, Voshol H, Meingassner JG, Mao X, Werner G, Rot A, Carballido JM (2008). Triggering the succinate receptor GPR91 on dendritic cells enhances immunity.. Nat Immunol.

[32] Tannahill GM (2013). Succinate is an inflammatory signal that induces IL-1beta through HIF-1alpha.. Nature.

[33] Schlame M, Greenberg ML (2017). Biosynthesis, remodeling and turnover of mitochondrial cardiolipin.. Biochim Biophys Acta.

[34] Tatsuta T, Langer T (2017). Intramitochondrial phospholipid trafficking.. Biochim Biophys Acta.

[35] Gebert N, Joshi AS, Kutik S, Becker T, McKenzie M, Guan XL, Mooga VP, Stroud DA, Kulkarni G, Wenk MR, Rehling P, Meisinger C, Ryan MT, Wiedemann N, Greenberg ML, Pfanner N (2009). Mitochondrial cardiolipin involved in outer-membrane protein biogenesis: implications for Barth syndrome.. Curr Biol.

[36] Maguire JJ, Tyurina YY, Mohammadyani D, Kapralov AA, Anthonymuthu TS, Qu F, Amoscato AA, Sparvero LJ, Tyurin VA, PlanasIglesias J, He R-R, Klein-Seetharaman J, Bayir H, Kagan VE (2017). Known unknowns of cardiolipin signaling: The best is yet to come.. Biochim Biophys Acta.

[37] Claypool SM, Koehler CM (2012). The complexity of cardiolipin in health and disease.. Trends Biochem Sci.

[38] Chicco AJ, Sparagna GC (2007). Role of cardiolipin alterations in mitochondrial dysfunction and disease.. Am J Physiol Cell Physiol.

[39] Dieude M, Striegl H, Tyznik AJ, Wang J, Behar SM, Piccirillo CA, Levine JS, Zajonc DM, Rauch J (2011). Cardiolipin binds to CD1d and stimulates CD1d-restricted gammadelta T cells in the normal murine repertoire.. J Immunol.

[40] Iyer SS, He Q, Janczy JR, Elliott EI, Zhong Z, Olivier AK, Sadler JJ, Knepper-Adrian V, Han R, Qiao L, Eisenbarth SC, Nauseef WM, Cassel SL, Sutterwala FS (2013). Mitochondrial cardiolipin is required for Nlrp3 inflammasome activation.. Immunity.

[41] Ray NB, Durairaj L, Chen BB, McVerry BJ, Ryan AJ, Donahoe M, Waltenbaugh AK, O'Donnell CP, Henderson FC, Etscheidt CA, McCoy DM, Agassandian M, Hayes-Rowan EC, Coon TA, Butler PL, Gakhar L, Mathur SN, Sieren JC, Tyurina YY, Kagan VE, McLennan G, Mallampalli RK (2010). Dynamic regulation of cardiolipin by the lipid pump Atp8b1 determines the severity of lung injury in experimental pneumonia.. Nat Med.

[42] Carp H (1982). Mitochondrial N-formylmethionyl proteins as chemoattractants for neutrophils.. J Exp Med.

[43] Le Y, Murphy PM, Wang JM (2002). Formyl-peptide receptors revisited.. Trends Immunol.

[44] Panaro MA, Acquafredda A, Sisto M, Lisi S, Maffione AB, Mitolo V (2006). Biological role of the N-formyl peptide receptors.. Immunopharmacol Immunotoxicol.

[45] Czapiga M, Gao J-L, Kirk A, Lekstrom-Himes J (2005). Human platelets exhibit chemotaxis using functional N-formyl peptide receptors.. Exp Hematol.

[46] Rabiet M-J, Huet E, Boulay F (2005). Human mitochondria-derived N-formylated peptides are novel agonists equally active on FPR and FPRL1, while Listeria monocytogenes-derived peptides preferentially activate FPR.. Eur J Immunol.

[47] Daniel MA, McDonald G, Offenbacher S, Van Dyke TE (1993). Defective chemotaxis and calcium response in localized juvenile periodontitis neutrophils.. J Periodontol.

[48] Van Dyke TE, Levine MJ, Tabak LA, Genco RJ (1981). Reduced chemotactic peptide binding in juvenile periodontitis: a model for neutrophil function.. Biochem Biophys Res Commun.

[49] Kozak M (1983). Comparison of initiation of protein synthesis in procaryotes, eucaryotes, and organelles.. Microbiol Rev.

[50] Gustafsson CM, Falkenberg M, Larsson N-G (2016). Maintenance and Expression of Mammalian Mitochondrial DNA.. Annu Rev Biochem.

[51] Kaufman B a, Newman SM, Hallberg RL, Slaughter C a, Perlman PS, Butow R a (2000). In organello formaldehyde crosslinking of proteins to mtDNA: identification of bifunctional proteins.. Proc Natl Acad Sci U S A.

[52] Ngo HB, Lovely GA, Phillips R, Chan DC (2014). Distinct structural features of TFAM drive mitochondrial DNA packaging versus transcriptional activation.. Nat Commun.

[53] Clayton DA (1982). Replication of animal mitochondrial DNA.. Cell.

[54] Bogenhagen DF (2012). Mitochondrial DNA nucleoid structure.. Biochim Biophys Acta - Gene Regul Mech.

[55] Larsson NG, Wang J, Wilhelmsson H, Oldfors a, Rustin P, Lewandoski M, Barsh GS, Clayton D a (1998). Mitochondrial transcription factor A is necessary for mtDNA maintenance and embryogenesis in mice.. Nat Genet.

[56] Kukat C, Davies KM, Wurm C a., Spåhr H, Bonekamp N a., Kühl I, Joos F, Polosa PL, Park CB, Posse V, Falkenberg M, Jakobs S, Kühlbrandt W, Larsson N-G (2015). Cross-strand binding of TFAM to a single mtDNA molecule forms the mitochondrial nucleoid.. Proc Natl Acad Sci.

[57] Brown TA, Tkachuk AN, Shtengel G, Kopek BG, Bogenhagen DF, Hess HF, Clayton DA (2011). Superresolution Fluorescence Imaging of Mitochondrial Nucleoids Reveals Their Spatial Range, Limits, and Membrane Interaction.. Mol Cell Biol.

[58] Kukat C, Wurm CA, Spåhr H, Falkenberg M, Larsson N (2011). Super-resolution microscopy reveals that mammalian mitochondrial nucleoids have a uniform size and frequently contain a single copy of mtDNA.. Proc Natl Acad Sci U S A.

[59] Anderson S, Bankier AT, Barrell BG, de Bruijn MH, Coulson AR, Drouin J, Eperon IC, Nierlich DP, Roe BA, Sanger F, Schreier PH, Smith AJ, Staden R, Young IG (1981). Sequence and organization of the human mitochondrial genome.. Nature.

[60] Clayton DA, Hughes H, Chase C (2000). Transcription and replication of mitochondrial DNA.. Hum Reprod.

[61] Oka T, Hikoso S, Yamaguchi O, Taneike M, Takeda T, Tamai T, Oyabu J, Murakawa T, Nakayama H, Nishida K, Akira S, Yamamoto A, Komuro I, Otsu K (2012). Mitochondrial DNA that escapes from autophagy causes inflammation and heart failure.. Nature.

[62] McCarthy CG, Wenceslau CF, Goulopoulou S, Ogbi S, Baban B, Sullivan JC, Matsumoto T, Webb RC (2015). Circulating mitochondrial DNA and Toll-like receptor 9 are associated with vascular dysfunction in spontaneously hypertensive rats.. Cardiovasc Res.

[63] Nakahira K, Haspel JA, Rathinam V a K, Lee S-J, Dolinay T, Lam HC, Englert J a, Rabinovitch M, Cernadas M, Kim HP, Fitzgerald K a, Ryter SW, Choi AMK (2011). Autophagy proteins regulate innate immune responses by inhibiting the release of mitochondrial DNA mediated by the NALP3 inflammasome.. Nat Immunol.

[64] West AP, Khoury-Hanold W, Staron M, Tal MC, Pineda CM, Lang SM, Bestwick M, Duguay BA, Raimundo N, MacDuff DA, Kaech SM, Smiley JR, Means RE, Iwasaki A, Shadel GS (2015). Mitochondrial DNA stress primes the antiviral innate immune response.. Nature.

[65] Picca A, LEZZA AMS, Leeuwenburgh C, PESCE V, Calvani R, Bossola M, Manes-Gravina E, Landi F, Bernabei R, Marzetti E (2017). Circulating mitochondrial DNA at the crossroads of mitochondrial dysfunction and inflammation during aging and muscle wasting disorders.. Rejuvenation Res.

[66] Hong EE, Okitsu CY, Smith AD, Hsieh C-L (2013). Regionally Specific and Genome-Wide Analyses Conclusively Demonstrate the Absence of CpG Methylation in Human Mitochondrial DNA.. Mol Cell Biol.

[67] Shock LS, Thakkar P V., Peterson EJ, Moran RG, Taylor SM (2011). DNA methyltransferase 1, cytosine methylation, and cytosine hydroxymethylation in mammalian mitochondria.. Proc Natl Acad Sci.

[68] Shimada K, Crother TR, Karlin J, Dagvadorj J, Chiba N, Chen S, Ramanujan VK, Wolf AJ, Vergnes L, Ojcius DM, Rentsendorj A, Vargas M, Guerrero C, Wang Y, Fitzgerald K a, Underhill DM, Town T, Arditi M (2012). Oxidized mitochondrial DNA activates the NLRP3 inflammasome during apoptosis.. Immunity.

[69] Collins LV, Hajizadeh S, Holme E, Jonsson I, Tarkowski A (2004). Endogenously oxidized mitochondrial DNA induces in vivo and in vitro inflammatory responses.. J Leukoc Biol.

[70] Tsuji N, Tsuji T, Ohashi N, Kato A, Fujigaki Y, Yasuda H (2015). Role of Mitochondrial DNA in Septic AKI via Toll-Like Receptor 9.. J Am Soc Nephrol.

[71] Marques PE, Amaral SS, Pires DA, Nogueira LL, Soriani FM, Lima BHF, Lopes GAO, Russo RC, Avila T V, Melgaco JG, Oliveira AG, Pinto MA, Lima CX, De Paula AM, Cara DC, Leite MF, Teixeira MM, Menezes GB (2012). Chemokines and mitochondrial products activate neutrophils to amplify organ injury during mouse acute liver failure.. Hepatology.

[72] Zhang L, Deng S, Zhao S, Ai Y, Zhang L, Pan P, Su X, Tan H, Wu D (2016). Intra-Peritoneal Administration of Mitochondrial DNA Provokes Acute Lung Injury and Systemic Inflammation via Toll-Like Receptor 9.. Int J Mol Sci.

[73] Zhang Q, Itagaki K, Hauser CJ (2010). Mitochondrial DNA is released by shock and activates neutrophils via p38 map kinase.. Shock.

[74] Wilkins HM, Koppel SJ, Weidling IW, Roy N, Ryan LN, Stanford JA, Swerdlow RH (2016). Extracellular Mitochondria and Mitochondrial Components Act as Damage-Associated Molecular Pattern Molecules in the Mouse Brain.. J Neuroimmune Pharmacol.

[75] Wilkins HM, Carl SM, Weber SG, Ramanujan SA, Festoff BW, Linseman DA, Swerdlow RH (2015). Mitochondrial Lysates Induce Inflammation and Alzheimer's Disease-Relevant Changes in Microglial and Neuronal Cells.. J Alzheimers Dis.

[76] Campbell CT, Kolesar JE, Kaufman BA (2012). Mitochondrial transcription factor A regulates mitochondrial transcription initiation, DNA packaging, and genome copy number.. Biochim Biophys Acta.

[77] Kang D, Kim SH, Hamasaki N (2007). Mitochondrial transcription factor A (TFAM): roles in maintenance of mtDNA and cellular functions.. Mitochondrion.

[78] Julian MW, Shao G, Bao S, Knoell DL, Papenfuss TL, VanGundy ZC, Crouser ED (2012). Mitochondrial transcription factor A serves as a danger signal by augmenting plasmacytoid dendritic cell responses to DNA.. J Immunol.

[79] Julian MW, Shao G, Vangundy ZC, Papenfuss TL, Crouser ED (2013). Mitochondrial transcription factor A, an endogenous danger signal, promotes TNFα release via RAGE- and TLR9-responsive plasmacytoid dendritic cells.. PLoS One.

[80] Crouser ED, Shao G, Julian MW, Macre JE, Shadel GS, Tridandapani, Susheela Huang Q, and D, Wewers M (2010). Monocyte Activation by Necrotic Cells Is Promoted by Mitochondrial Proteins and Formyl Peptide Receptors Elliott.. Crit Care Med.

[81] Chaung WW, Wu R, Ji Y, Dong W, Wang P (2012). Mitochondrial transcription factor A is a proinflammatory mediator in hemorrhagic shock.. Int J Mol Med.

[82] Zhang Q, Raoof M, Chen Y, Sumi Y, Sursal T, Junger W, Brohi K, Itagaki K, Hauser CJ (2010). Circulating mitochondrial DAMPs cause inflammatory responses to injury.. Nature.

[83] Hu Q, Wood CR, Cimen S, Venkatachalam AB, Alwayn IPJ (2015). Mitochondrial damage-associated molecular patterns (MTDs) are released during hepatic ischemia reperfusion and induce inflammatory responses.. PLoS One.

[84] Schoenauer R, Atanassoff AP, Wolfmeier H, Pelegrin P, Babiychuk EB, Draeger A (2014). P2X7 receptors mediate resistance to toxininduced cell lysis.. Biochim Biophys Acta - Mol Cell Res.

[85] Duregotti E, Negro S, Scorzeto M, Zornetta I, Dickinson BC, Chang CJ, Montecucco C, Rigoni M (2015). Mitochondrial alarmins released by degenerating motor axon terminals activate perisynaptic Schwann cells.. Proc Natl Acad Sci.

[86] Liu J, Epand RF, Durrant D, Grossman D, Chi N, Epand RM, Lee RM (2008). Role of phospholipid scramblase 3 in the regulation of tumor necrosis factor-alpha-induced apoptosis.. Biochemistry.

[87] Chu CT, Ji J, Dagda RK, Jiang JF, Tyurina YY, Kapralov AA, Tyurin VA, Yanamala N, Shrivastava IH, Mohammadyani D, Wang KZQ, Zhu J, Klein-Seetharaman J, Balasubramanian K, Amoscato AA, Borisenko G, Huang Z, Gusdon AM, Cheikhi A, Steer EK, Wang R, Baty C, Watkins S, Bahar I, Bayir H, Kagan VE (2013). Cardiolipin externalization to the outer mitochondrial membrane acts as an elimination signal for mitophagy in neuronal cells.. Nat Cell Biol.

[88] Iyer SS, He Q, Janczy JR, Elliott EI, Zhong Z, Olivier AK, Sadler JJ, Knepper-Adrian V, Han R, Qiao L, Eisenbarth SC, Nauseef WM, Cassel SL, Sutterwala FS (2013). Mitochondrial cardiolipin is required for Nlrp3 inflammasome activation.. Immunity.

[89] Sun S, Sursal T, Adibnia Y, Zhao C, Zheng Y, Li H, Otterbein LE, Hauser CJ, Itagaki K (2013). Mitochondrial DAMPs increase endothelial permeability through neutrophil dependent and independent pathways.. PLoS One.

[90] He W, Miao FJ-P, Lin DC-H, Schwandner RT, Wang Z, Gao J, Chen J-L, Tian H, Ling L (2004). Citric acid cycle intermediates as ligands for orphan G-protein-coupled receptors.. Nature.

[91] Migeotte I, Communi D, Parmentier M (2006). Formyl peptide receptors: a promiscuous subfamily of G protein-coupled receptors controlling immune responses.. Cytokine Growth Factor Rev.

[92] West AP, Shadel GS (2017). Mitochondrial DNA in innate immune responses and inflammatory pathology.. Nat Rev Immunol.

[93] Takeuchi O, Akira S (2010). Pattern recognition receptors and inflammation.. Cell.

[94] Wu J, Sun L, Chen X, Du F, Shi H, Chen C, Chen ZJ (2013). Cyclic GMP-AMP is an endogenous second messenger in innate immune signaling by cytosolic DNA.. Science.

[95] Sun L, Wu J, Du F, Chen X, Chen ZJ (2012). Cyclic GMP-AMP Synthase Is a Cytosolic DNA Sensor That Activates the Type I Interferon Pathway.. Science.

[96] Cai D (2009). NFkappaB-mediated metabolic inflammation in peripheral tissues versus central nervous system.. Cell Cycle.

[97] Ishii KJ, Coban C, Kato H, Takahashi K, Torii Y, Takeshita F, Ludwig H, Sutter G, Suzuki K, Hemmi H, Sato S, Yamamoto M, Uematsu S, Kawai T, Takeuchi O, Akira S (2006). A Toll-like receptor-independent antiviral response induced by double-stranded B-form DNA.. Nat Immunol.

[98] Ishikawa H, Barber GN (2008). STING is an endoplasmic reticulum adaptor that facilitates innate immune signalling.. Nature.

[99] Stetson DB, Medzhitov R (2006). Recognition of cytosolic DNA activates an IRF3-dependent innate immune response.. Immunity.

[100] Ishikawa H, Ma Z, Barber GN (2009). STING regulates intracellular DNA-mediated, type I interferon-dependent innate immunity.. Nature.

[101] Zhong B, Yang Y, Li S, Wang Y-Y, Li Y, Diao F, Lei C, He X, Zhang L, Tien P, Shu H-B (2008). The adaptor protein MITA links virus-sensing receptors to IRF3 transcription factor activation.. Immunity.

[102] Lei Z, Deng M, Yi Z, Sun Q, Shapiro RA, Xu H, Li T, Loughran PA, Griepentrog JE, Huang H, Scott MJ, Huang F, Billiar TR (2018). cGAS-mediated autophagy protects the liver from ischemia/reperfusion injury independent of STING.. Am J Physiol Liver Physiol.

[103] Andreeva L, Hiller B, Kostrewa D, Lässig C, de Oliveira Mann CC, Jan Drexler D, Maiser A, Gaidt M, Leonhardt H, Hornung V, Hopfner K-P (2017). cGAS senses long and HMGB/TFAM-bound U-turn DNA by forming protein–DNA ladders.. Nature.

[104] Bai J, Cervantes C, Liu J, He S, Zhou H, Zhang B, Cai H, Yin D, Hu D, Li Z, Chen H, Gao X, Wang F, O'Connor JC, Xu Y, Liu M, Dong LQ, Liu F (2017). DsbA-L prevents obesity-induced inflammation and insulin resistance by suppressing the mtDNA release-activated cGAS-cGAMP-STING pathway.. Proc Natl Acad Sci.

[105] McArthur K, Whitehead LW, Heddleston JM, Li L, Padman BS, Oorschot V, Geoghegan ND, Chappaz S, Davidson S, Chin HS, Lane RM, Dramicanin M, Saunders TL, Sugiana C, Lessene R, Osellame LD, Chew TL, Dewson G, Lazarou M, Ramm G, Lessene G, Ryan MT, Rogers KL, Van Delft MF, Kile BT (2018). BAK/BAX macropores facilitate mitochondrial herniation and mtDNA efflux during apoptosis.. Science.

[106] Kim G-T, Cho M-L, Park Y-E, Yoo WH, Kim J-H, Oh H-J, Kim D-S, Baek S-H, Lee S-H, Lee J-H, Kim H-Y, Kim S-I (2010). Expression of TLR2, TLR4, and TLR9 in dermatomyositis and polymyositis.. Clin Rheumatol.

[107] Barbalat R, Ewald SE, Mouchess ML, Barton GM (2011). Nucleic acid recognition by the innate immune system.. Annu Rev Immunol.

[108] Latz E, Schoenemeyer A, Visintin A, Fitzgerald KA, Monks BG, Knetter CF, Lien E, Nilsen NJ, Espevik T, Golenbock DT (2004). TLR9 signals after translocating from the ER to CpG DNA in the lysosome.. Nat Immunol.

[109] Lamphier MS, Sirois CM, Verma A, Golenbock DT, Latz E (2006). TLR9 and the recognition of self and non-self nucleic acids.. Ann N Y Acad Sci.

[110] Leifer CA, Kennedy MN, Mazzoni A, Lee C, Kruhlak MJ, Segal DM (2004). TLR9 is localized in the endoplasmic reticulum prior to stimulation.. J Immunol.

[111] Ewald SE, Lee BL, Lau L, Wickliffe KE, Shi GP, Chapman HA, Barton GM (2008). The ectodomain of Toll-like receptor 9 is cleaved to generate a functional receptor.. Nature.

[112] Chockalingam A, Brooks JC, Cameron JL, Blum LK, Leifer CA (2009). TLR9 traffics through the Golgi complex to localize to endolysosomes and respond to CpG DNA.. Immunol Cell Biol.

[113] Tabeta K, Hoebe K, Janssen EM, Du X, Georgel P, Crozat K, Mudd S, Mann N, Sovath S, Goode J, Shamel L, Herskovits AA, Portnoy DA, Cooke M, Tarantino LM, Wiltshire T, Steinberg BE, Grinstein S, Beutler B (2006). The Unc93b1 mutation 3d disrupts exogenous antigen presentation and signaling via Toll-like receptors 3, 7 and 9.. Nat Immunol.

[114] Fukui R, Saitoh S, Matsumoto F, Kozuka-Hata H, Oyama M, Tabeta K, Beutler B, Miyake K (2009). Unc93B1 biases Toll-like receptor responses to nucleic acid in dendritic cells toward DNA-but against RNA-sensing.. J Exp Med.

[115] Pelka K, Phulphagar K, Zimmermann J, Stahl R, Schmid-Burgk JL, Schmidt T, Spille J-H, Labzin LI, Agrawal S, Kandimalla ER, Casanova J-L, Hornung V, Marshak-Rothstein A, Höning S, Latz E (2014). Cutting edge: the UNC93B1 tyrosine-based motif regulates trafficking and TLR responses via separate mechanisms.. J Immunol.

[116] Hayashi K, Sasai M, Iwasaki A (2015). Toll-like receptor 9 trafficking and signaling for type i interferons requires PIKfyve activity.. Int Immunol.

[117] Honda K, Ohba Y, Yanai H, Negishi H, Mizutani T, Takaoka A, Taya C, Taniguchi T (2005). Spatiotemporal regulation of MyD88-IRF-7 signalling for robust type-I interferon induction.. Nature.

[118] Lee BL, Barton GM (2014). Trafficking of endosomal Toll-like receptors.. Trends Cell Biol.

[119] Duhamel M, Rodet F, Murgoci AN, Desjardins R, Gagnon H, Wisztorski M, Fournier I, Day R, Salzet M (2016). The proprotein convertase PC1/3 regulates TLR9 trafficking and the associated signaling pathways.. Sci Rep.

[120] Shintani Y, Kapoor A, Kaneko M, Smolenski RT, D'Acquisto F, Coppen SR, Harada-Shoji N, Lee HJ, Thiemermann C, Takashima S, Yashiro K, Suzuki K (2013). TLR9 mediates cellular protection by modulating energy metabolism in cardiomyocytes and neurons.. Proc Natl Acad Sci U S A.

[121] Shintani Y, Drexler HC, Kioka H, Terracciano CM, Coppen SR, Imamura H, Akao M, Nakai J, Wheeler AP, Higo S, Nakayama H, Takashima S, Yashiro K, Suzuki K (2014). Toll-like receptor 9 protects non-immune cells from stress by modulating mitochondrial ATP synthesis through the inhibition of SERCA2.. EMBO Rep.

[122] Hajizadeh S, DeGroot J, TeKoppele JM, Tarkowski A, Collins LV (2003). Extracellular mitochondrial DNA and oxidatively damaged DNA in synovial fluid of patients with rheumatoid arthritis.. Arthritis Res Ther.

[123] Ding Z, Liu S, Wang X, Khaidakov M, Dai Y, Mehta JL (2013). Oxidant stress in mitochondrial DNA damage, autophagy and inflammation in atherosclerosis.. Sci Rep.

[124] Yao X, Carlson D, Sun Y, Ma L, Wolf SE, Minei JP, Zang QS (2015). Mitochondrial ROS Induces Cardiac Inflammation via a Pathway through mtDNA Damage in a Pneumonia-Related Sepsis Model.. PLoS One.

[125] Zhang L, Deng S, Zhao S, Ai Y, Zhang L, Pan P, Su X, Tan H, Wu D (2016). Intra-peritoneal administration of mitochondrial DNA provokes acute lung injury and systemic inflammation via toll-like receptor 9.. Int J Mol Sci.

[126] Li Y, Berke IC, Modis Y (2012). DNA binding to proteolytically activated TLR9 is sequence-independent and enhanced by DNA curvature.. EMBO J.

[127] Zhang JZ, Liu Z, Liu J, Ren JX, Sun TS (2014). Mitochondrial DNA induces inflammation and increases TLR9/NF-κB expression in lung tissue.. Int J Mol Med.

[128] Bao W, Xia H, Liang Y, Ye Y, Lu Y, Xu X, Duan A, He J, Chen Z, Wu Y, Wang X, Zheng C, Liu Z, Shi S (2016). Toll-like Receptor 9 Can be Activated by Endogenous Mitochondrial DNA to Induce Podocyte Apoptosis.. Sci Rep.

[129] Garcia-martinez I, Santoro N, Chen Y, Hoque R, Ouyang X, Caprio S, Shlomchik MJ, Coffman RL, Candia A, Mehal WZ (2016). Hepatocyte mitochondrial DNA drives nonalcoholic steatohepatitis by activation of TLR9.. J Clin Invest.

[130] Kuck JL, Obiako BO, Gorodnya OM, Pastukh VM, Kua J, Simmons JD, Gillespie MN (2015). Mitochondrial DNA damage-associated molecular patterns mediate a feed-forward cycle of bacteria-induced vascular injury in perfused rat lungs.. Am J Physiol - Lung Cell Mol Physiol.

[131] Goulopoulou S, Matsumoto T, Bomfim GF, Webb RC (2012). Toll-like receptor 9 activation: a novel mechanism linking placenta-derived mitochondrial DNA and vascular dysfunction in pre-eclampsia.. Clin Sci.

[132] Tian J, Avalos AM, Mao S-Y, Chen B, Senthil K, Wu H, Parroche P, Drabic S, Golenbock D, Sirois C, Hua J, An LL, Audoly L, La Rosa G, Bierhaus A, Naworth P, Marshak-Rothstein A, Crow MK, Fitzgerald K a, Latz E, Kiener P a, Coyle AJ (2007). Toll-like receptor 9-dependent activation by DNA-containing immune complexes is mediated by HMGB1 and RAGE.. Nat Immunol.

[133] Julian MW, Shao G, Bao S, Knoell DL, Papenfuss TL, Vangundy ZC, Crouser ED, Daren L, Papenfuss TL, Vangundy ZC, Crouser ED (2012). Mitochondrial transcription factor A serves as a danger signal by augmenting plasmacytoid dendritic cell responses to DNA.. J Immunol.

[134] Leo MG De, Staiano L, Vicinanza M, Luciani A, Carissimo A, Mutarelli M, Campli A Di, Polishchuk E, Tullio G Di, Morra V, Levtchenko E, Oltrabella F, Starborg T, Santoro M, Bernardo D, Devuyst O, Lowe M, Medina DL, Ballabio A, Matteis MA De (2016). Autophagosome – lysosome fusion triggers a lysosomal response mediated by TLR9 and controlled by OCRL.. Nat Cell Biol.

[135] Rodríguez-Nuevo A, Díaz-Ramos A, Noguera E, Díaz-Sáez F, Duran X, Muñoz JP, Romero M, Plana N, Sebastián D, Tezze C, Romanello V, Ribas F, Seco J, Planet E, Doctrow SR, González J, Borràs M, Liesa M, Palacín M, Vendrell J, Villarroya F, Sandri M, Shirihai O, Zorzano A (2018). Mitochondrial DNA and TLR9 drive muscle inflammation upon Opa1 deficiency.. EMBO J.

[136] Liu Y, Yan W, Tohme S, Chen M, Fu Y, Tian D, Lotze M, Tang D, Tsung A (2015). Hypoxia induced HMGB1 and mitochondrial DNA interactions mediate tumor growth in hepatocellular carcinoma through Toll Like Receptor 9.. J Hepatol.

[137] Green NM, Marshak-Rothstein A (2011). Toll-like receptor driven B cell activation in the induction of systemic autoimmunity.. Semin Immunol.

[138] Santiago-Raber ML, Baudino L, Izui S (2009). Emerging roles of TLR7 and TLR9 in murine SLE.. J Autoimmun.

[139] Lamphier M, Zheng W, Latz E, Spyvee M, Hansen H, Rose J, Genest M, Yang H, Shaffer C, Zhao Y, Shen Y, Liu C, Liu D, Mempel TR, Rowbottom C, Chow J, Twine NC, Yu M, Gusovsky F, Ishizaka ST (2014). Novel small molecule inhibitors of TLR7 and TLR9: mechanism of action and efficacy in vivo.. Mol Pharmacol.

[140] Broz P, Dixit VM (2016). Inflammasomes: mechanism of assembly, regulation and signalling.. Nat Rev Immunol.

[141] Man SM, Kanneganti T-D (2015). Converging roles of caspases in inflammasome activation, cell death and innate immunity.. Nat Rev Immunol.

[142] Place DE, Kanneganti TD (2018). Recent advances in inflammasome biology.. Curr Opin Immunol.

[143] Kawaguchi M, Takahashi M, Hata T, Kashima Y, Usui F, Morimoto H, Izawa A, Takahashi Y, Masumoto J, Koyama J, Hongo M, Noda T, Nakayama J, Sagara J, Taniguchi S, Ikeda U (2011). Inflammasome activation of cardiac fibroblasts is essential for myocardial ischemia/reperfusion injury.. Circulation.

[144] Wree A, Eguchi A, McGeough MD, Pena C a, Johnson CD, Canbay A, Hoffman HM, Feldstein AE (2013). NLRP3 inflammasome activation results in hepatocyte pyroptosis, liver inflammation and fibrosis.. Hepatology.

[145] Boaru SG, Borkham-Kamphorst E, Van de Leur E, Lehnen E, Liedtke C, Weiskirchen R (2015). NLRP3 inflammasome expression is driven by NF-κB in cultured hepatocytes.. Biochem Biophys Res Commun.

[146] Kanneganti TD (2015). The inflammasome: Firing up innate immunity.. Immunol Rev.

[147] Shahzad K, Bock F, Dong W, Wang H, Kopf S, Kohli S, Al-Dabet MM, Ranjan S, Wolter J, Wacker C, Biemann R, Stoyanov S, Reymann K, Söderkvist P, Groß O, Schwenger V, Pahernik S, Nawroth PP, Gröne H-J, Madhusudhan T, Isermann B (2014). Nlrp3-inflammasome activation in non-myeloid-derived cells aggravates diabetic nephropathy.. Kidney Int.

[148] Zhuang Y, Yasinta M, Hu C, Zhao M, Ding G, Bai M, Yang L, Ni J, Wang R, Jia Z, Huang S, Zhang A (2015). Mitochondrial dysfunction confers albumin-induced NLRP3 inflammasome activation and renal tubular injury.. Am J Physiol - Ren Physiol.

[149] Rawat R, Cohen T V, Ampong B, Francia D, Henriques-Pons A, Hoffman EP, Nagaraju K (2010). Inflammasome up-regulation and activation in dysferlin-deficient skeletal muscle.. Am J Pathol.

